# Role of LOXL2 in the epithelial-mesenchymal transition and colorectal cancer metastasis

**DOI:** 10.18632/oncotarget.18170

**Published:** 2017-05-25

**Authors:** Pil-Gu Park, Su Ji Jo, Min Jung Kim, Hyun Jeong Kim, Ji Hae Lee, Cheol Keun Park, Hyunki Kim, Kang Young Lee, Hoguen Kim, Jeon Han Park, Seung Myung Dong, Jae Myun Lee

**Affiliations:** ^1^ Department of Microbiology and Immunology, Yonsei University College of Medicine, Seoul, Republic of Korea; ^2^ BK21 PLUS Project for Medical Science, Yonsei University College of Medicine, Seoul, Republic of Korea; ^3^ Department of Pediatrics, Severance Hospital, Institute of Allergy, Yonsei University College of Medicine, Seoul, Republic of Korea; ^4^ Department of Nuclear Medicine, Yonsei University College of Medicine, Seoul, Republic of Korea; ^5^ Research Institute, National Cancer Center, Goyang, Republic of Korea; ^6^ Department of Pathology, Yonsei University, College of Medicine, Seoul, South Korea; ^7^ Department of Surgery, Severance Hospital, Yonsei University College of Medicine, Seoul, Republic of Korea; ^8^ IMK Bio-Convergence R&D Center, International Vaccine Institute SNU Research Park, Seoul, Republic of Korea

**Keywords:** colorectal cancer, metastasis, LOXL2, epithelial-mesenchymal transition, tissue microarray analysis

## Abstract

Colorectal cancer (CRC) is one of the most dangerous types of malignant tumors, and cancer metastasis is a major factor in the failure of CRC therapy. Recently, LOXL2 (lysyl oxidase-like 2) has been shown to represent a regulator of epithelial-mesenchymal transition (EMT) in different cancer types. However, LOXL2 has not been reported to be involved in CRC metastasis. In this study, we demonstrated that LOXL2 expression is strongly correlated with the rate of CRC metastasis, it participates in the regulation of EMT-related molecule expression in CRC cells *in vitro*, and it is involved in migratory potential alterations. Additionally, tissue microarray analysis of CRC patients showed an increase in the probability of developing CRC distant metastasis and a decrease in the survival rate of patients with high *LOXL2* expression. The results obtained in this study indicate that LOXL2 is involved in the development and progression of CRC metastasis, and therefore, its expression levels may represent a useful prognostic marker.

## INTRODUCTION

Colorectal cancer (CRC) is the third most common cancer worldwide (∼10% of all cancer cases) and the second most prevalent cancer (2.4 million people) [1, 2]. Approximately 700,000 people yearly die of CRC, and the development of metastatic disease is the most frequent cause of death [1, 3, 4]. The majority of CRC patients is diagnosed at the advanced cancer stage, and the risk of metastatic progression is increased in these patients [5]. Therefore, further studies should focus on the molecular mechanisms underlying the development of CRC metastasis, which may lead to the development of improved treatment strategies.

Epithelial-mesenchymal transition (EMT) is a molecular process essential for cancer metastasis initiation. During the process of EMT, cell properties are altered, and those required for migration, invasion into the extracellular matrix (ECM), and extravasation/intravasation, are acquired [6, 7]. In addition to these changes, the changes in the levels of many molecules can be observed, including several regulatory molecules that modulate the EMT.

Lysyl oxidase-like 2 (LOXL2) is a member of lysyl oxidase (LOX) family, which includes molecules sharing a highly conserved catalytic domain at the C-terminus [8]. In physiological conditions, LOXL2 is involved in the extracellular matrix (ECM) remodeling. Similar to the other members of LOX family, LOXL2 induces the crosslinking of ECM collagen and elastin by the deamination of peptidyl lysine residues [8, 9]. Recently, several groups reported that LOXL2 may represent a modulator of EMT, through the regulation of EMT-inducing transcription factors, focal adhesion kinase (FAK)/Src signaling pathway, and the epigenetic regulation of EMT-related genes [10–12].

Furthermore, many studies, including ours, investigated and showed the clinical relevance of LOXL2 expression during the metastasis of breast [13], gastric [14], liver [15], esophageal [16], and pancreatic [17] cancers. However, LOXL2 in CRC has been rarely investigated [18, 19], and these studies focused on LOXL2 expression in association with CRC survival rate and tumor differentiation, and, to the best of our knowledge, the correlation between LOXL2 expression and the development of CRC metastasis has not been previously investigated. However, since the loss of the heterozygosity (LOH) locus is frequently found in patients with colon cancer metastasis, and LOXL2 was reported to be one of the genes located at this locus [20], this may indicate a correlation between the expression levels of this molecule and the development of CRC metastasis. Therefore, we investigated the role of LOXL2 in the development and progression of CRC metastasis both *in vitro* and in patients.

## RESULTS

### CRC cell migratory potential is associated with LOXL2 expression

**Figure 1:**
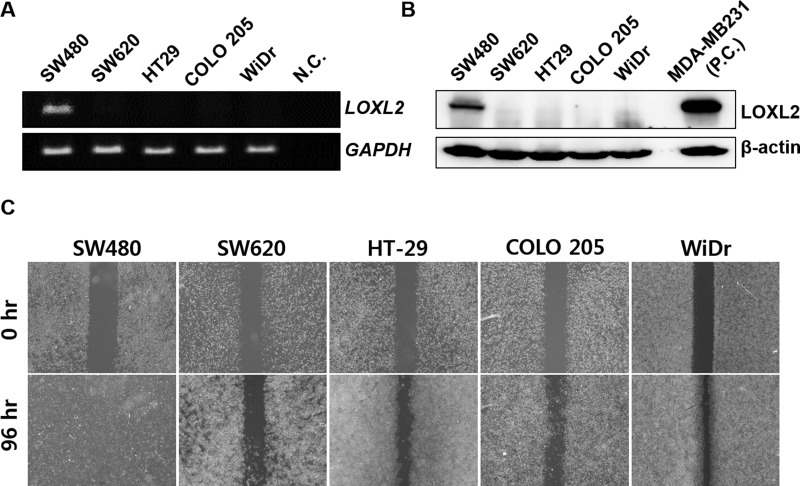
Correlation between LOXL2 expression levels and migratory potential of different CRC cells *in vitro* (**A**) *LOXL2* expression in five CRC cell lines. Distilled water was used as a negative control (N.C.). (**B**) LOXL2 protein levels in CRC cells. MDA-MB-231 was used as a positive control (P.C.). (**C**) Motility of different CRC cells *in vitro,* determined in wound healing assay. Representative figures obtained in three independent experiment are shown.

We first examined LOXL2 expression levels in several CRC cell lines. LOXL2 was shown to be expressed in SW480 cells, but rarely detected in other investigated cell lines, at mRNA and protein levels (Figure 1A and 1B). Although the expression of LOXL2 was determined to be lower than that determined in MDA-MB-231 cells, LOXL2-positive breast cancer cells [13], SW480 cells showed considerably higher migratory potential than LOXL2-negative cells, in migration assay and wound healing assay (Figure 1C and Supplementary Figure 1A). Cell migration rate was shown to be independent of the growth rate of different cells (Supplementary Figure 1B).

**Figure 2:**
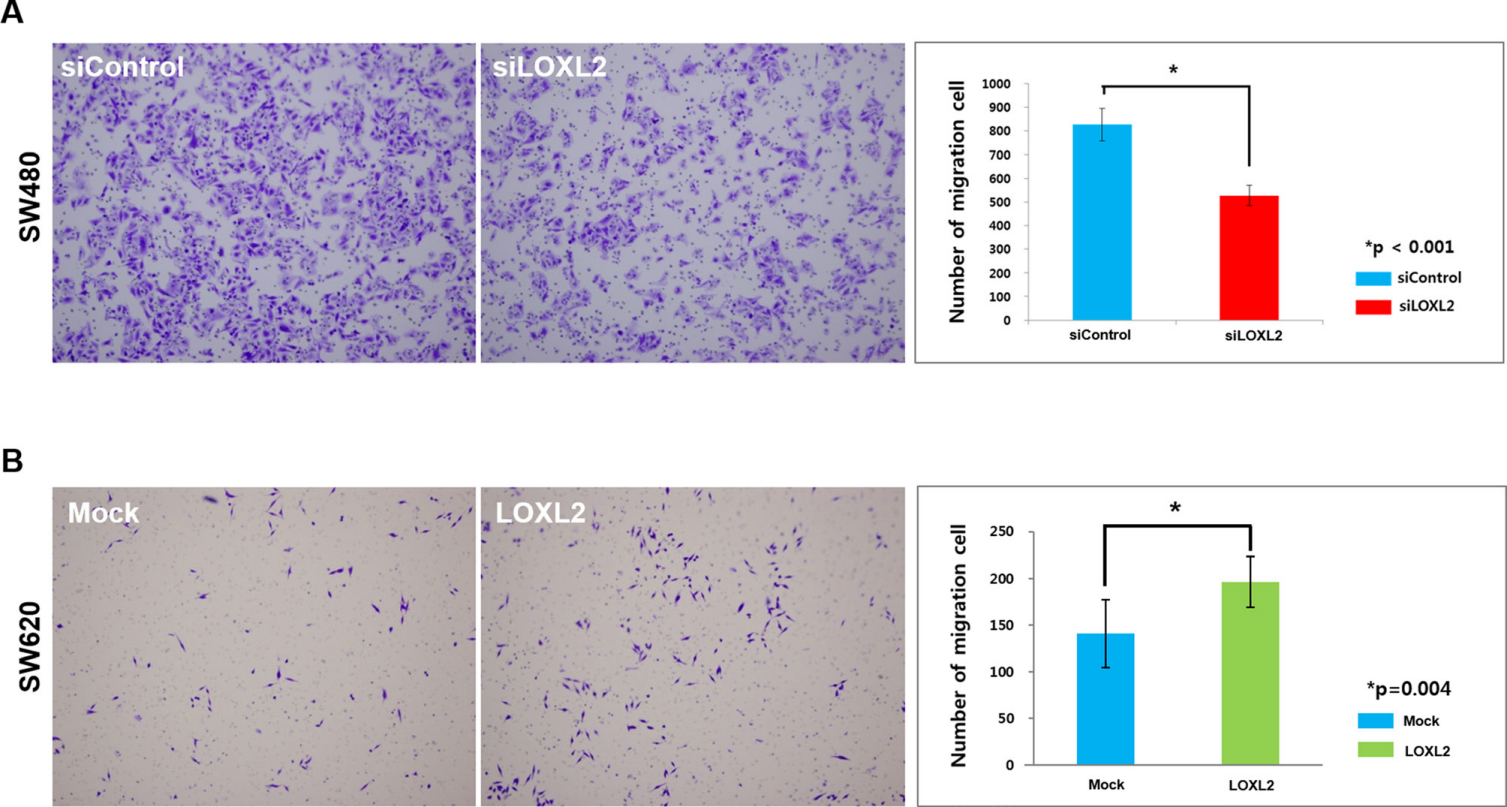
Migratory potential alterations induced by the changes in *LOXL2* expression (**A**) Transwell migration of *LOXL2*-knockdown SW480 cells. Images were obtained 36 h after seeding. (**B**) Transwell migration of *LOXL2*-overexpressing SW620 cells. Images were obtained 72 h after seeding. Results were obtained from three independent experiments, and bar graphs in (A) and (B) represent cell number per image field (mean ± standard deviation).

Next, we examined the migratory phenotype by using *LOXL2* knockdown and ectopic expression in SW480 and SW620 (LOXL2-negative) cells, respectively. We demonstrated that *LOXL2* knockdown led to a significant reduction in the migratory potential of SW480 cells (Figure 2A and Supplementary Figure 2), while its ectopic expression led to the stimulation of SW620 cell migratory potential (Figure 2B).

**Figure 3:**
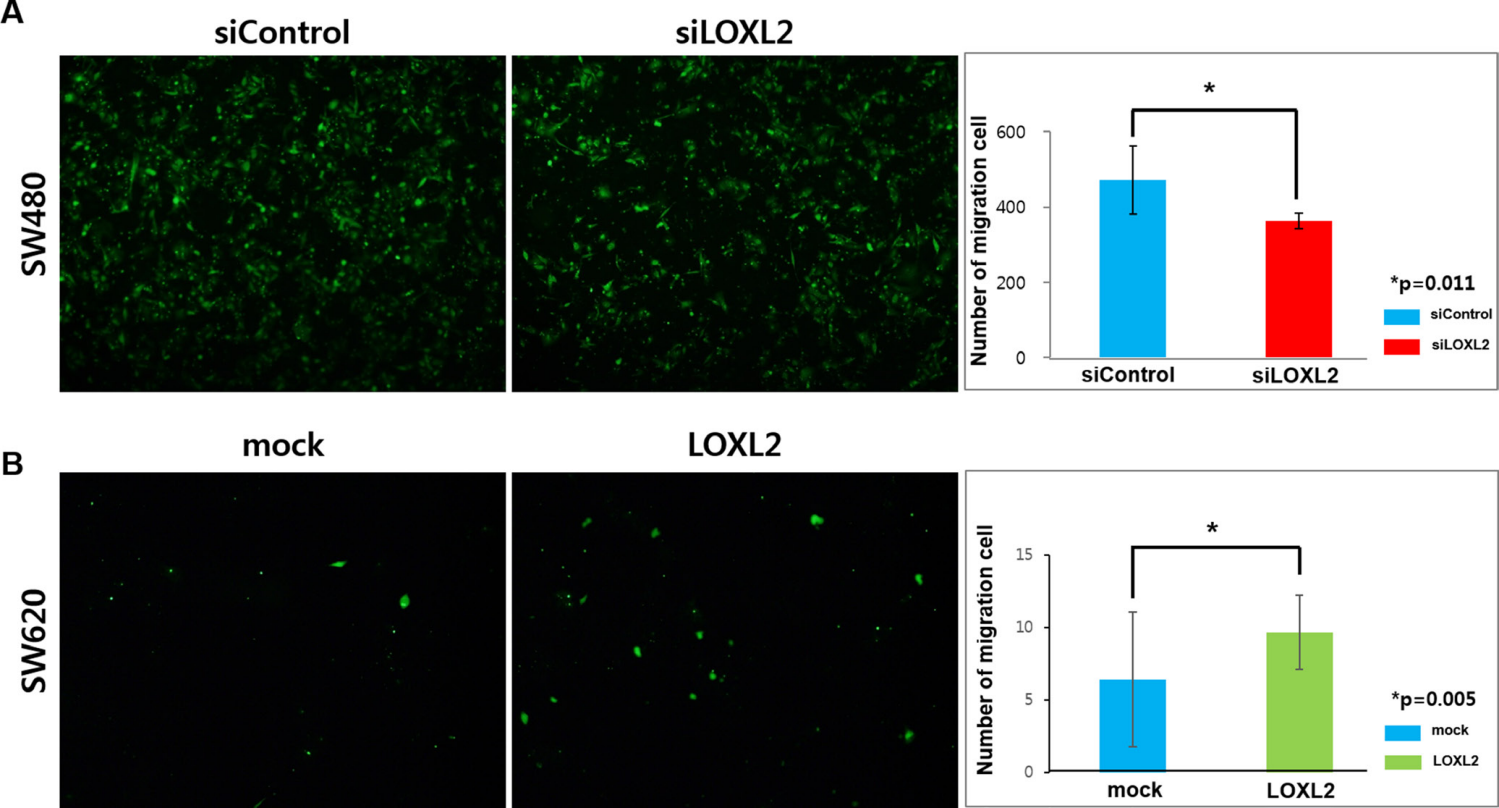
Effects of *LOXL2* silencing/ectopic expression on the migration of CRC cells through the endothelial barrier (**A**) SW480 cells transfected with siControl/siLOXL2 oligonucleotide were seeded on HUVECs, and images were obtained 36 h after seeding. (**B**) SW620 cells transfected with mock/*LOXL2*-expressing plasmids were seeded on HUVECs, and images were obtained 72 h after seeding. Results were obtained from three independent experiments and bar graphs in (A) and (B) represent cell number per image field (mean ± standard deviation).

Furthermore, we examined the ability of these cells to cross the endothelial cell barrier, represented by human umbilical vein endothelial cells (HUVEC), which mimics intra/extravasation process during metastasis. We observed that many SW480 cells were able to penetrate HUVEC cell layer, but *LOXL2* silencing led to a decrease in this potential (Figure 3A). SW620 cells transfected with the *LOXL2* expression plasmid showed an increase in the HUVEC layer penetration, compared with that of the cells transfected with a mock plasmid (Figure 3B). Additionally, we confirmed that *LOXL2* knockdown and overexpression did not affect cellular growth rates (Supplementary Figure 3).

### LOXL2 regulates the expression of EMT-related molecules in CRC cells

**Figure 4:**
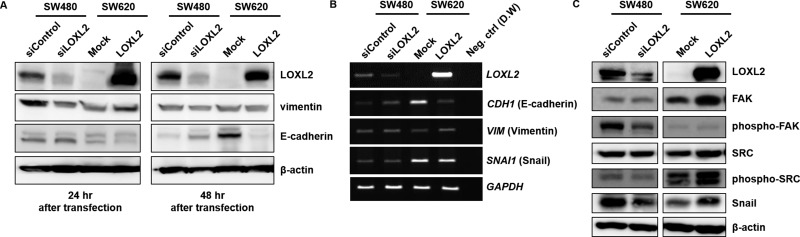
Induction of EMT by LOXL2 through the activation of FAK/Src pathway and upregulation of Snail (**A**) Expression of EMT-related molecules 24 and 48 h after the transfection of cells with LOXL2 siRNA/overexpressing plasmid. (**B**) *CDH1* (E-cadherin), *VIM* (vimentin), and *SNAI1* (Snail) expression in SW480 and SW620 cells 24 h after the transfection with siLOXL2 and *LOXL2*-overexpressing plasmid, respectively. *GAPDH* was used as an internal control. (**C**) Expression of EMT-related molecules in cells transfected with siLOXL2 and *LOXL2*-overexpressing plasmid.

In order to investigate the molecular mechanism underlying LOXL2-dependent alterations in migratory potential, we examined *LOXL2* knockdown/ectopic expression-induced alterations in SW480 and SW620 cells, respectively (Figure 4A). We showed that *LOXL2* knockdown leads to a decrease in the expression of vimentin, a mesenchymal marker, while *LOXL2* overexpression upregulated the expression of this protein. Conversely, we obtained opposite effects on the expression of the epithelial marker, E-cadherin. Similar results were obtained at mRNA level (Figure 4B).

Furthermore, since the activation of FAK/Src pathway is known to promote EMT, several reports indicated that the changes in ECM stiffness can lead to the activation of FAK/Src signaling [21–23], and LOXL2 is known to be involved in the ECM remodeling, we investigated whether LOXL2 is involved in the activation of FAK/Src by determining the levels of phosphorylated FAK/Src (Figure 4C). *LOXL2* knockdown and overexpression were shown to down- and upregulate, respectively, FAK/Src phosphorylation. Snail protein, one of the major transcription factors involved in the EMT process, was shown to be regulated by LOXL2, and *LOXL2* knockdown led to a decrease, while its overexpression led to an increase in Snail levels (Figure 4C).

### Correlation between LOXL2 expression levels and clinicopathological characteristics of CRC patients

**Figure 5:**
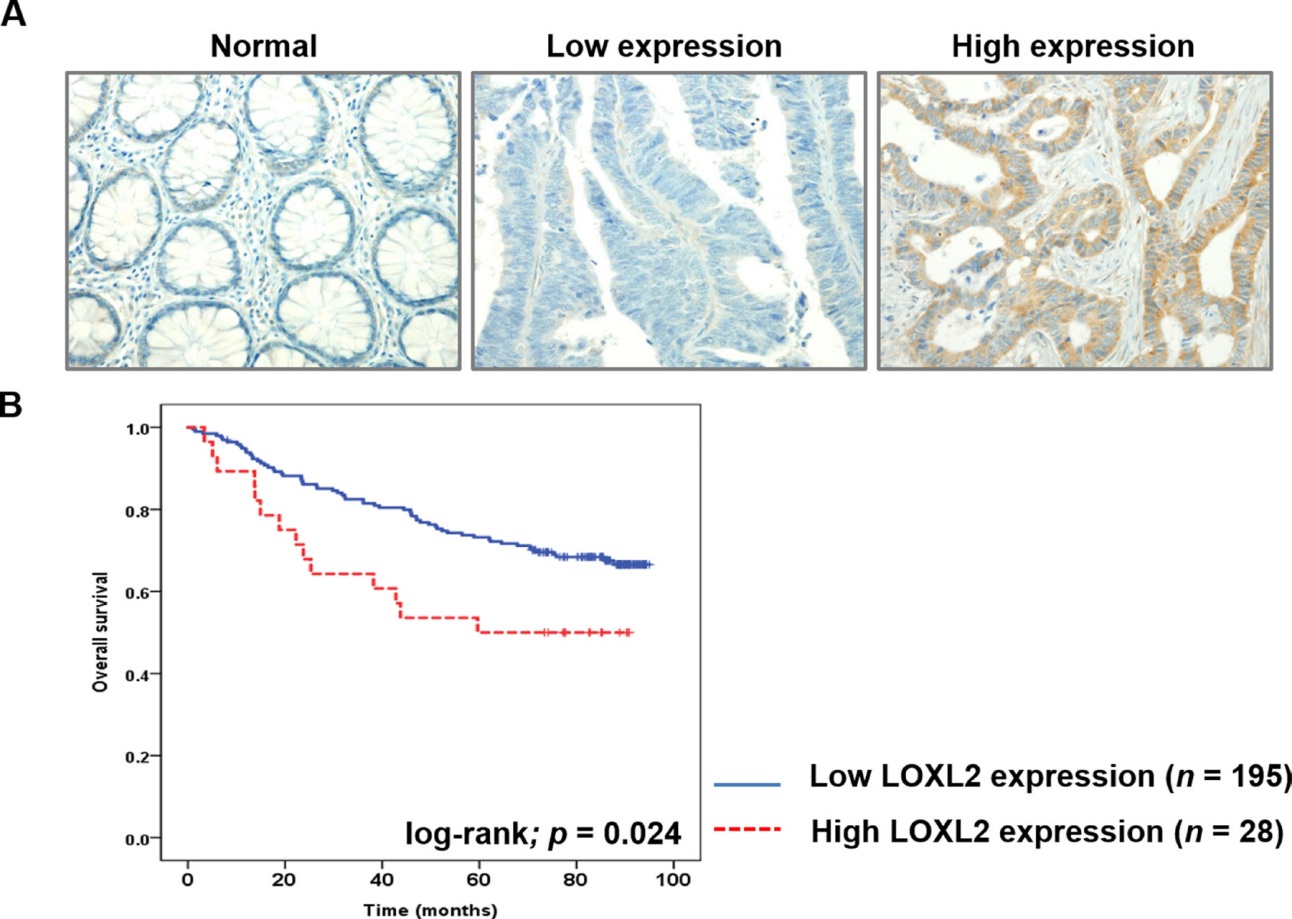
LOXL2 IHC analysis in CRC tissues and the correlation between this parameter and the survival of CRC patients (**A**) IHC analysis of LOXL2 expression in CRC tissues. Representative images of normal colon tissue, CRC tissue with low LOXL2 expression, and CRC tissue with high LOXL2 expression are presented (magnification, 200 ×). (**B**) Correlation between the overall survival rates of CRC patients and *LOXL2* expression. High LOXL2 expression was correlated with poor patient survival (log-rank Mantel-Cox test, *p* = 0.024).

We investigated the correlation between LOXL2 expression levels and CRC metastasis rate in patients, by immunohistochemically (IHC) examining LOXL2 expression in cancer tissue samples obtained from CRC patients. LOXL2 expression was classified as low or high (Figure 5A), and, of 223 patients examined in our study, the samples obtained from 28 patients (12.1%) were shown to have high LOXL2 expression levels, which was shown to be significantly associated with the presence of distant metastasis (*p* = 0.046). Increased LOXL2 expression levels tended to be associated with the survival of the patients (*p* = 0.066). Other clinicopathological parameters, including patient age, sex, histological grade, pathologic T stage, overall cancer stage, and the presence of lymph node metastasis were not associated with LOXL2 expression (*p* > 0.05 for all). Clinicopathological characteristics in patients categorized by LOXL2 expression levels are summarized in Table 1. Patients with high LOXL2 expression levels were shown to have a shorter overall survival (*p* = 0.024; Figure 5B) than those with low LOXL2 expression.

**Table 1:** Clinicopathological characteristics of CRC patients categorized based on their LOXL2 expression levels

Category	Variables	No. of cases (*n* = 223)	High expression (%) (*n* = 28)		Low expression (%) (*n* = 195)		*p*-value
Age	< 60	97	12	(42.9)	85	(43.6)	0.942
	≥ 60	126	16	(57.1)	110	(56.4)	
Gender	Female	81	13	(46.4)	68	(34.9)	0.235
	Male	142	15	(53.6)	127	(65.1)	
Location	Colon	112	13	(46.4)	99	(50.8)	0.668
	Rectum	111	15	(53.6)	96	(49.2)	
Histologic grade	WD to MD	205	25	(89.3)	180	(92.3)	0.584
	PD	18	3	(10.7)	15	(7.7)	
Pathologic T stage	T1 and T2	30	3	(10.7)	27	(13.8)	0.650
	T3 and T4	193	25	(89.3)	168	(86.2)	
LNM	Absent	103	11	(39.3)	92	(47.2)	0.434
	Present	120	17	(60.7)	103	(52.8)	
Distant metastasis	Absent	188	20	(71.4)	168	(86.2)	0.046
	Present	35	8	(28.6)	27	(13.8)	
Overall cancer stage	I	23	1	(3.6)	22	(11.3)	0.104
	II	73	9	(32.1)	64	(32.8)	
	III	92	10	(35.7)	82	(42.1)	
	IV	35	8	(28.6)	27	(13.8)	
Survival	Alive	146	14	(50.0)	132	(67.7)	0.066
	Expired	77	14	(50.0)	63	(32.3)	

WD: well differentiated; MD: moderately differentiated; PD: poorly differentiated; LNM: lymph node metastasis.

## DISCUSSION

Here, we investigated the role of LOXL2 in the EMT progression in CRC, and clinical consequences of the changes in LOXL2 expression. We observed that LOXL2-positive CRC cells have an increased migratory potential, in comparison with that of the LOXL2-negative cells; *LOXL2* knockdown/ectopic expression was shown to affect this migratory potential in these cells *in vitro*, demonstrating that LOXL2 promotes the EMT process, while the results obtained by analyzing clinicopathological parameters strongly support the pro-metastatic role of LOXL2 in CRC progression.

Various molecular mechanisms underlying LOXL2-dependent EMT induction have been reported. Peinado *et al*. [10] demonstrated that LOXL2 stabilizes Snail, one of the major transcription factors inducing EMT process, by oxidizing lysine 98 and 137 residues, and this process and the phosphorylation of Snail by glycogen synthase kinase 3 beta (GSK3β), which is a signal for proteasome-dependent degradation, are considered antagonistic processes. Here, we demonstrated that the changes in *LOXL2* expression affect Snail at protein, but not at mRNA level, suggesting that *LOXL2* modulates SNAI1 at the post-translation levels, which further affects the migratory potential of CRC cells *in vitro*.

Other members of LOX family have been suggested to induce the activation of EMT, including LOX, which was reported to be involved in the development of CRC metastasis [24], through the activation of FAK/Src signaling pathway, which induces the EMT process, and the activation of the pathway induced by ECM remodeling, which may trigger the activation of cellular receptors, such as integrin. Previously, several studies reported that LOXL2 induces EMT in breast, gastric, and pancreatic cancer cells, which is accompanied by the activation of FAK/Src pathway [13, 14, 17]. This may suggest that all members of LOX family induce EMT through ECM remodeling. However, LOXL2 may play the key role in this process, because this is the only member of LOX family that can stabilize Snail [10]. Here, we observed that LOXL2 induces FAK/Src activation and regulates Snail *in vitro*, and therefore, we suggest that LOXL2 may have dual effects on the EMT activation in CRC cells.

Metastatic cells generally have a higher migratory potential compared with that of the primary tumor cells. SW480 and SW620 cell lines have originated from one patient. The former was isolated from a primary colorectal cancer and the latter from a lymph node metastasis [25]. However, the results obtained here demonstrated that SW480 cells show higher motility and migratory potential than SW620 *in vitro*. Previously, different studies reported controversial results when analyzing migratory potential of these cells [25–35], which may be due to the alterations in cell line characteristic during long-term *in vitro* culturing, or it is possible that SW620 cells underwent mesenchymal-epithelial transition (MET), in order to establish and grow at the distant metastatic sites [36, 37].

In spite of inverted migratory capacity to conceptual expectation, it is possible that SW620 possesses higher metastatic potential than SW480 *in vivo*. It was reported in several studies that SW620 cells show an increased metastatic potential in xenograft experiments compared with that of SW480 cells [38, 39]. Due to the complexity of metastatic process, other factors, such as proteolytic potential and cancer cell stemness are crucial for this process as well [6, 40], and some of these factors may be responsible for the observed discrepancy between the migratory capacity and metastatic potential of two analyzed cell lines. Although high migratory potential is not sufficient for the development of metastasis, it is necessary, and therefore, our results strongly indicate that LOXL2 plays crucial role in the development of CRC metastasis.

Although previous reports suggested that LOXL2 expression may be related to CRC differentiation and stage [18, 19], the results of our clinical analysis showed a correlation between CRC metastasis rate and LOXL2 expression, which agrees with the results obtained in studies investigating LOXL2 roles in other cancer types [13–17], and the study examining the LOH locus in CRC patients with liver metastasis [20]. Among 13 genes at the LOH locus, *LOXL2* expression was shown to be increased in patients with liver metastasis, compared with that in the normal tissues or matched primary cancer by locus mutation, which supports our conclusion that LOXL2 induces EMT/metastasis. Furthermore, LOXL2 expression was shown to be associated with patient survival rate, suggesting that the levels of this molecule may serve as a prognostic marker in CRC.

Currently, different characteristics are used in clinic for the classification of CRCs. Microsatellite instability (MSI), one of the essential factors used for the classification of CRCs, occurs due to the deficiencies in mismatch repair (MMR) mechanism, believed to represent a main cause of carcinogenesis [41, 42]. Clinically, five to eight specific loci in CRC patients are investigated in order to establish the diagnosis of MSI- or microsatellite stable (MSS)-CRC, and this process is crucial for the selection of therapeutic strategies [43]. While it is believed that MSI-CRCs have a more positive prognosis and lower frequency of metastasis [44], 80∼85% of CRCs are diagnosed as MSS-CRCs [45], requiring further molecular studies of the mechanism of carcinogenesis and metastasis process. Here, we used MSS-CRC cells, since only six patients (2.7%) were diagnosed as MSI-CRC in TMA analysis performed in this study (data not shown), but all screened cell lines were of MSS-CRC origin.

In conclusion, the results obtained in our study demonstrated that LOXL2 expression levels may be of clinical significance in CRC, and the mechanisms underlying the effects of this molecule include the activation of FAK/Src signaling and the stabilization of Snail. The analysis of clinicopathological parameters of CRC patients demonstrated that LOXL2 may represent a valuable prognostic marker for these patients. Furthermore, molecular mechanisms underlying LOXL2-mediated EMT induction in CRC, which we elucidated here, may lead to the development of improved therapeutic strategies for the treatment of CRC metastasis. Targeting of transcription factors or signaling molecules associated with the process of EMT may generally have harmful effects, due to the presence and functions of these molecules in the healthy tissues. However, LOXL2 is generally not expressed in normal colon tissues [46], which may allow a specific targeting of CRC metastatic cells, and, since LOXL2 is an enzyme, its potential inhibitors can be easily and rapidly screened. The clinical application of several inhibitors targeting LOXL2 is being investigated currently, although not for the treatment of CRC [47, 48]. Taken together, our results and previous reports demonstrate that LOXL2 may represent a promising therapeutic target for the treatment of metastatic CRC.

## MATERIALS AND METHODS

### Cell culture

All CRC cell lines (SW480, SW620, HT-29, COLO 205, and WiDr) were purchased from the American Type Culture Collection (ATCC, Manassas, VA, USA), and cultured in Dulbecco's Modified Eagle Medium (DMEM; Hyclone, Logan, UT, USA) supplemented with 10% fetal bovine serum (FBS; Hyclone) and 10 U/mL of penicillin-streptomycin (Hyclone). All cell lines were routinely tested for mycoplasma contamination and cell lines used in subsequent experiments (SW480 and SW620) were authenticated by short tandem repeat profiling at the Research Institute of National Cancer Center (Republic of Korea).

HUVEC cells were purchased from ATCC, and cultured in Endothelial Cell Growth medium (EGM-2; Lonza, Walkersville, MD, USA) supplemented with 10% FBS.

### Knockdown and ectopic expression of LOXL2

For *LOXL2* knockdown, cells were transfected with small interfering (si)RNA targeting *LOXL2* (siLOXL2; 5′-GCCACAUAGGUGGUUCCUUCAUU-3′) or non specific oligonucleotide (siControl; 5′-CGUUAAUCG CGUAUAAUACGCGUA-3′) using Lipofectamine RNAiMAX (Invitrogen/Life Technologies, Green Island, NY, USA), according to the manufacturer's instructions.

For the ectopic expression of *LOXL2*, cells were transfected with *LOXL2*-expressing plasmid (pcDNA3.1(+)−hLOXL2 [17]) using Lipofectamine 2000 (Invitrogen/Life Technologies) according to the manufacturer's instructions.

### Reverse transcription-polymerase chain reaction (RT-PCR)

Total RNA was extracted from CRC cells using TRIZOL reagent (Gibco BRL/Life Technologies, Green Island, NY, USA), and cDNA was subsequently synthesized using SuperScriptIII First-Strand Synthesis System (Invitrogen/Life Technologies), according to the manufacturer's instructions. Synthesized cDNAs were used as a template for subsequent PCR, determining the levels of *SNAI1*, *LOXL2*, *VIM, CDH1,* and *GAPDH*. Primer sequences used in each PCR set are shown in Supplementary Table 1.

### Western blot analysis

Cells were lysed with radioimmunoprecipitation assay (RIPA) buffer (Sigma-Aldrich, St. Louis, MO, USA) supplemented with protease inhibitors and sampled in protein sample buffer (250 mM Tris-Cl, 1.6 mM EDTA, 8% sodium dodecyl sulfate (SDS), 40% glycerol, 0.02% Pyronin Y dye, 1.5% 2′-mercaptoethanol). Protein samples were separated using 8–10% SDS-polyacrylamide gel electrophoresis and transferred onto nitrocellulose membranes, which were blocked and probed with antibodies against LOXL2 (Origene, Rockville, MD, USA), SNAI1 (Cell Signaling, Danvers, MA, USA), phosphor-FAK;Tyr576, and FAK (Santa Cruz Biotechnology, CA, USA), phosphor-Src;Tyr418 (Millipore, Billerica, MA, USA), Src (Santa Cruz Biotechnology), CDH1 (BD Biosciences, Sparks, MD), VIM (Dako, Glostrup, Denmark), and β-actin (Sigma-Aldrich). The blots were prepared using WesternBright Sirius solution (Advansta; Menlo Park, CA, USA) and analyzed with enhanced chemiluminescence using ImageQuant LAS Chemi-luminescent Image Analyzer (GE Healthcare/Life Technologies, Green Island, NY, USA).

### Wound-healing assay

In order to induce a wound in a monolayer of CRC cells, a culture insert (IBIDI, Martinsried, Germany), consisting of two reservoirs separated by a 500-μm thick wall, was placed into each well of six-well plates, and 5 × 10^4^ cells were added into the two reservoirs. After 24 h of incubation at 37°C, the insert was gently removed, and the cells were allowed to migrate for 5 days. Images were taken every 24 h using Olympus IX71 inverted microscope equipped with a digital camera (Olympus France, Rungis, France).

For the comparative analysis of migratory potential according to *LOXL2* expression in SW480 cells, control SW480 cells and *LOXL2*-silenced SW480 cells (5 × 10^4^ cells/well) were seeded into 96-well Essen ImageLock microplates (Essen BioScience, Ann Arbor, MI, USA), and incubated at 37°C for 18 h. Cell monolayers were scratched using a 96-well Wound Maker (Essen BioScience), and the wells were washed with phosphate-buffered saline (PBS) to remove the detached cells. Images of the wounds were automatically recorded at every 6 h for 96 h using IncuCyte ZOOM software (Essen BioScience).

### Transwell migration and trans-endothelial migration assays

The migratory capacity of CRC cells was assessed *in vitro* using transwell migration chambers (Corning, NY, USA) in accordance with the manufacturer's instructions. Briefly, cells were serum-starved for 24 h, then detached by brief trypsinization, and resuspended in the serum-free medium. For each well, 2–4 × 10^5^ cells were seeded onto 0.1% gelatin-coated insert in the transwell chamber (8-μm pore size and 6.5-mm diameter), and the inserts were set in chambers filled with 400 μL of complete medium. Cells were incubated in a CO_2_ incubator at 37°C for 36 or 72 h, and the inserts were fixed with methanol and stained with hematoxylin-eosin solution (Sigma-Aldrich). Following the staining, the upper surface of membranes, containing the remaining non-migrated cells, was wiped with cotton swabs, and the lower membrane surfaces, containing cells that migrated, were mounted onto slides. Images were taken using Olympus IX71 inverted microscope equipped with a digital camera (Olympus, France).

For trans-endothelial migration assay, 3 × 10^4^ HUVECs were seeded onto 0.1% gelatin-coated insert 24 h prior to the seeding of 2–4 × 10^5^ carboxyfluorescein succinimidyl ester-labeled (CFSE; Thermo Fisher, Waltham, MA, USA) cells resuspended in serum-free medium. The inserts were set in the wells filled with the complete medium. After 36 or 72 h of incubation, the upper surface of the membranes was wiped with cotton swabs and the migrated cells on the lower surface fixed and mounted onto slides. Images were taken using Olympus DP71 fluorescent microscope equipped with a digital camera (Olympus France, Rungis, France) with green fluorescent protein (GFP) filter, in order to visualize CFSE-labeled cells.

All experiments were performed three times and, in each experiment, images were taken in 4–5 random fields per slide at 100× magnification.

### Cell proliferation assay

For the comparison of proliferation rates between five investigated CRC cell lines, 5 × 10^4^ cells of each line were seeded into 12-well plates and cell numbers were manually determined using disposable hemocytometers every 24 h.

For the proliferation assay with *LOXL2*-knockdown/overexpression cells, Cell Counting Kit-8 (Dojindo Molecular Technologies Inc, Rockville, MD, USA) was used, according to the manufacturer's instructions. Relative proliferation rates were calculated by detecting absorbance at 450 nm using Epoch Microplate Spectrophotometer (BioTek Instruments Inc, Winooski, VT, USA).

### CRC patient selection and tissue microarray construction

Patients who underwent surgical resection due to CRC in 2003 and 2004 at Severance Hospital (Seoul, Republic of Korea) were included in this study. Patients treated with neoadjuvant chemoradiation therapy, which may affect the EMT process [49], were excluded. Various clinicopathologic factors, such as the age at operation, gender, size and location of tumor, and clinical follow-up data were obtained from the review of medical records. Overall survival time was estimated from the date of curative resection to the date of the last follow-up or death from any cause.

Pathologic factors, such as histologic grade of tumor and pathologic TNM staging according to the 7^th^ American Joint Committee on Cancer criteria were obtained from the slides independently reviewed by two pathologists (C. K. Park and H. Kim). Two cores of representative tumor area and one core of the representative normal area were extracted from each case for tissue microarray construction as previously described [13]. This study was approved by the Institutional Review Board of Severance Hospital, Seoul, Republic of Korea (IRB 4-2012-0026), and the study was conducted according to the principles expressed in the Declaration of Helsinki.

### IHC staining and analysis

Four-micrometer large tissue sections from the tissue microarray recipient blocks were used for IHC, which was performed using the Ventana Discovery XT automated staining system (Ventana Medical Systems, Inc., Tucson, AZ, USA) and anti-LOXL2 antibody (Origene).

Immunostained slides were evaluated by two pathologists (C. K. Park and H. Kim) independently. LOXL2 expression was categorized as negative, focal positive, and positive. Further, these samples were considered as low expression samples if they were negative or focal positive, while positive samples were considered high expression samples.

### Statistical analysis

For transmigration assay and trans-endothelial migration assay, statistical analyses and the determination of *p*-values were performed by using Student's *t*-test, with IBM SPSS Statistics software v. 23 (SPSS Inc., Chicago, IL, USA).

For the analysis of clinicopathological characteristics, SPSS for Windows version 21.0 (SPSS Inc.) was used. Mann-Whitney *U*-test and Fisher's exact test were used for the analysis of various clinicopathologic factors. *p*-values < 0.05 were considered statistically significant. Kaplan-Meier survival curves with log-rank statistics were applied to evaluate time to survival.

## SUPPLEMENTARY MATERIALS FIGURES AND TABLE



## Abbreviations

CRC: colorectal cancer
EMT: epithelial-mesenchymal transition
ECM: extracellular matrix
FAK: focal adhesion kinase
GSK3β: glycogen synthase kinase 3 beta
HUVEC: human umbilical vein endothelial cell
IHC: immunohistochemistry
LOH: loss of the heterozygosity
LOX: lysyl oxidase
LOXL2: lysyl oxidase-like 2
