# Supplementary material for: Role of LOXL2 in the epithelial-mesenchymal transition and colorectal cancer metastasis
Source: Oncotarget. 2017 May 25;8(46):80325–35. doi: 10.18632/oncotarget.18170 (PMC5655201; doi:10.18632/oncotarget.18170)
Supplement: Supplementary file 1 [file oncotarget-08-80325-s001.pdf]

Role of LOXL2 in the epithelial-mesenchymal transition and colorectal cancer metastasis

SUPPLEMENTARY MATERIALS

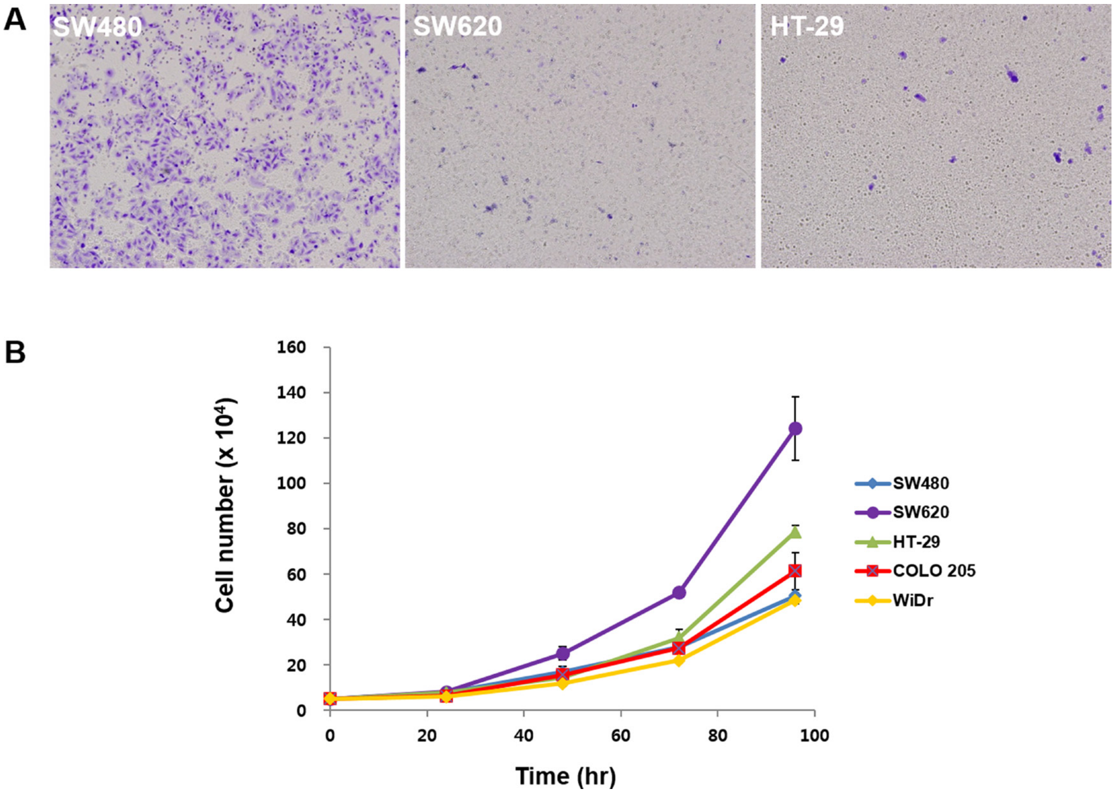

Supplementary Figure 1: Migratory potential of different CRC cells *in vitro*. (**A**) Representative images of *LOXL2*-positive CRC cell (SW480) and two *LOXL2*-negative cell (SW620 and HT-29) migration, obtained using transwell migration assay. (**B**) CRC cell proliferation. No correlation between cell proliferation rate and *LOXL2* expression levels was observed. All results in (A and B) were obtained in three independent experiments and data presented in (B) are presented as mean ± standard deviation.

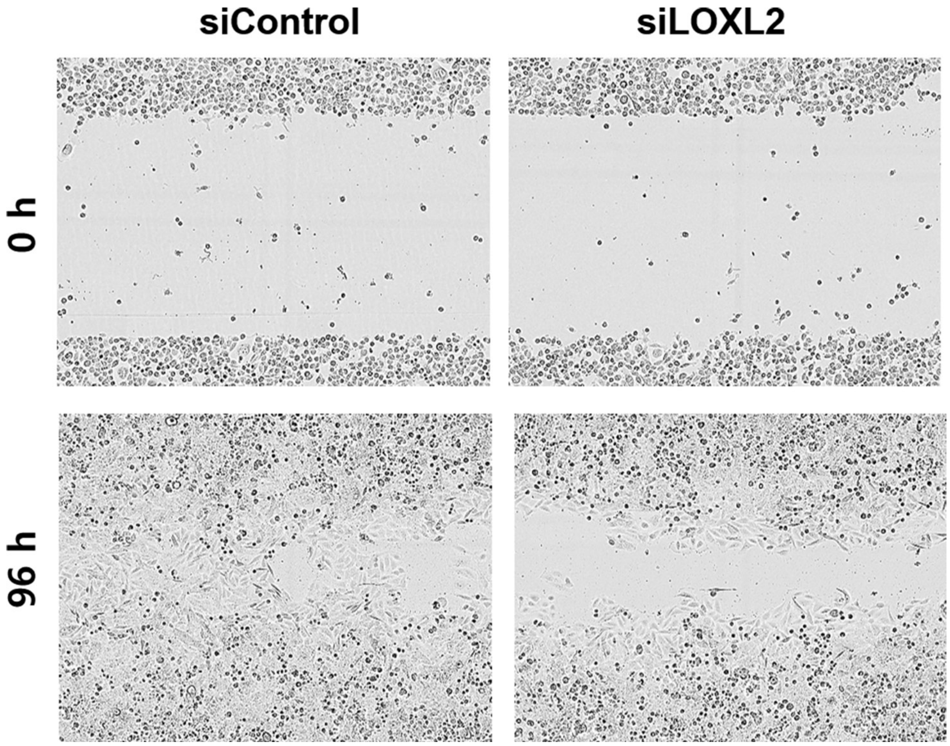

Supplementary Figure 2: Migratory potential of different CRC cells *in vitro*. Representative images obtained in the wound healing assay using SW480 cells with/without *LOXL2*-knockdown. Images obtained immediately and 96 h after wound-making are presented. The result were obtained in three independent experiments.

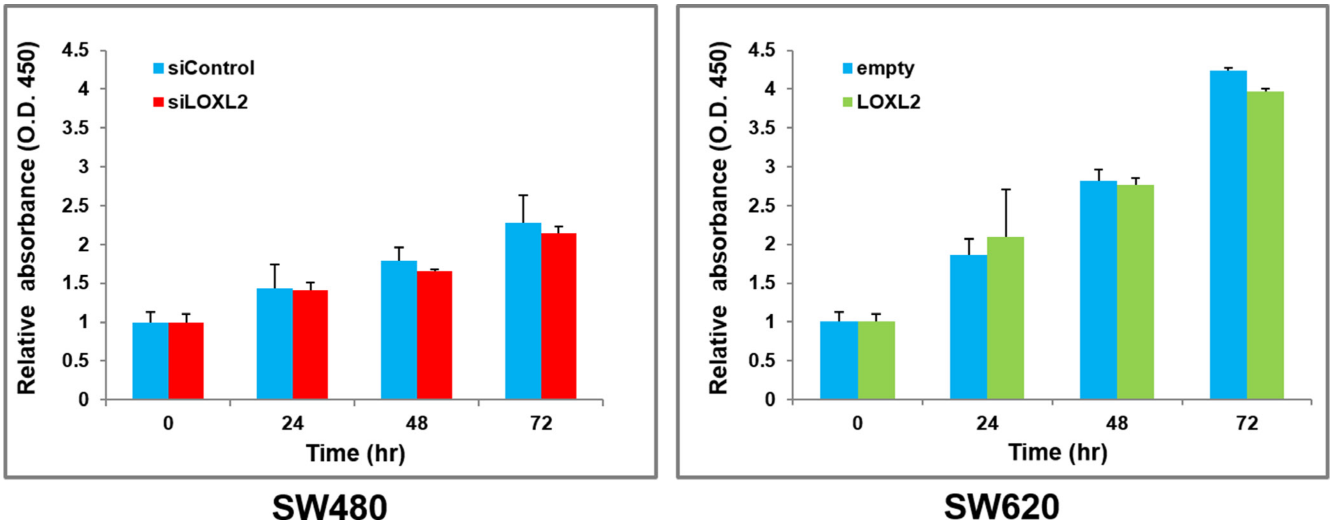

Supplementary Figure 3: SW480 and SW620 cell proliferation after the silencing or overexpression of *LOXL2*. Cell counting kit was used to determine cell proliferation rates. Data are presented as mean ± standard deviation obtained in three independent experiments.

Supplementary Table 1: Sequence of the primers used for RT-PCR analyses

Gene product	Sense	Antisense
LOXL2	AAAATGTTTGCC TCACGG	ACCAGGTCCCACTTGTCG
SNAI1 (Snail)	ACTTCAGTCTCTTCCTTGGAG	TGACATCTGAGTGGGTCTGG
CDH1 (E-cadherin)	GTCAGTTCAGACTCCAGCCC	AAATTCACTCTGCCCAGGACG
VIM (vimentin)	GTTTCCAAGCCTGACCTCAC	GCTTCAACGGCAAAGTTCTC
GAPDH	GATGGCATGGACTGTGGTCA	GCAATGCCTCCTGCACCACC
